# Supplementary material for: Critical Periods, Critical Time Points and Day-of-the-Week Effects in COVID-19 Surveillance Data: An Example in Middlesex County, Massachusetts, USA
Source: Int J Environ Res Public Health. 2022 Jan 25;19(3):1321. doi: 10.3390/ijerph19031321 (PMC8835321; doi:10.3390/ijerph19031321)
Supplement: Supplementary file 1 [file ijerph-19-01321-s001.zip › COVID_DoW_Supplementary_20211225.pdf]

Supplementary Material

Critical periods, critical time points and day-of-the-week effects in COVID-19 surveillance data: an example in Middlesex County, Massachusetts, USA

Ryan B. Simpson [1,a], Brianna N. Lauren [1,a], Kees H. Schipper [1], James C. McCann [1], Maia C. Tarnas [1], and Elena N. Naumova [1,*]

[1] Division of Nutrition Epidemiology and Data Science, Tufts University Friedman School of Nutrition Science and Policy, 150 Harrison Avenue, Boston, MA 02111, USA; brianna.lauren@tufts.edu; ryan.simpson@tufts.edu; kschipper0430@comcast.net; james.mccann@tufts.edu; maia.tarnas@gmail.com; elena.naumova@tufts.edu

a ryan.simpson@tufts.edu and brianna.lauren@tufts.edu had equal contribution as the first author.

* Correspondence: elena.naumova@tufts.edu; Tel.: 617-636-2927

Supplementary Table S1. Non-pharmaceutical intervention phases in accordance with Massachusetts state and Middlesex County, Massachusetts COVID-19 health advisories identified from numerous sources [S1-S72]. We provide start dates for each mandate and a brief explanation of the mandate required. We divide mandates by statewide announcements (complementing non-pharmaceutical intervention phases reported in Table 1), Middlesex County testing policies, vaccination-related policies, and county-level university and secondary school closures.

Start Date	Description
	Statewide Non-Pharmaceutical Interventions
24 March 2020	MA stay-at-home (SAH) advisory. Nonessential businesses ordered to close physical workplaces, and restaurants and bars restricted to offering takeout and delivery.
06 May 2020	Face coverings mandated in public places where social distancing is not possible
18 May 2020	MA SAH advisory changed to a "safer-at-home" advisory
01 August 2020	MA visitors returning from out of state required to fill out a form and quarantine for two weeks.
07 August 2020	Gov. Baker announced postponement of Phase III-2 reopening, intensifying enforcement of COVID-19 regulation, and reducing number of people allowed at private outdoor events from 100 to 50.
05 October 2020	Gov. Baker announced that 'lower risk' communities would be allowed to move into Phase III-2 of reopening, increasing indoor and outdoor performance venues to 50% capacity, up to 250 people. Fitting rooms could open in retail stores, and gyms, museums, libraries, and driving and flight schools could increase capacity to 50%.
22 October 2020	MADPH closing indoor ice rinks following clusters of COVID-19 at various rinks
02 November 2020	Gov. Baker announced statewide curfew for businesses and tighter limit on number of people allowed to gather indoors, and stricter face mask requirements. Indoor businesses must close by 9:30 pm and require restaurants to stop providing table services at the same time.
06 November 2020	Stay-at-home advisory initiated between the hours of 10 pm and 5 am, face coverings required everywhere, 9:30pm closure time for certain businesses
26 December 2020	25% capacity limit for businesses and activities implemented
25 January 2021	"Health clubs, movie theaters, casinos and more" able to stay open past 9:30pm
	Middlesex County COVID-19 Testing Policies
28 April 2020	Somerville begins offering free COVID-19 testing to all residents
10 July 2020	Massachusetts begins "Stop the Spread" testing initiative
	State and County COVID-19 Vaccination-Related Policies
11 January 2021	First responders to begin receiving first doses of the vaccine
18 January 2021	Gillette Stadium opens as mass vaccination site
29 January 2021	Fenway Park and Eastfield Mall mass vaccination clinics open
01 February 2021	Phase-II of vaccine program making residents older than 75 eligible for the vaccine, opening a mass vaccination site at Fenway Park.
02 February 2021	Reggie Lewis Center opens as mass vaccination clinic
03 February 2021	Doubletree Hotel mass vaccination clinic opens
22 February 2021	Natick Mall mass vaccination clinic opens
24 February 2021	Old Circuit City mass vaccination clinic opens
11 March 2021	Teachers and daycare providers eligible to sign up for appointments to receive the vaccine

22 March 2021	Hynes Convention Center mass vaccination site opens and is fully operational
County-Level School Closures	
07 March 2020	University Spring Break periods ranging from start dates of March 7th to March 20th
15 March 2020	K-12 schools closed statewide
18 March 2020	Childcare programs closed statewide, and emergency childcare programs created
20 April 2020	Spring break for public high schools in Middlesex county
07 May 2020	Universities in Middlesex County begin summer break with start dates ranging from May 7th to May 20th
16 May 2020	Summer break begins for public high schools in Middlesex county with start dates ranging from June 15th to June 18th
26 June 2020	Childcare facilities authorized for reopening statewide
10 August 2020	Universities in Middlesex County begin term with start dates ranging from Aug 10th to Sep 8th
15 August 2020	College of the Holy Cross counts 20 positive COVID-19 cases among students after an off-campus party
16 August 2020	Boston University and Clark University begin moving students into on-campus housing
31 August 2020	Term begins for public high schools in Middlesex county with start dates of Aug 31st to Sept 17th
04 September 2020	Four students dismissed without reimbursement of tuition and housing from Northeastern University
22 September 2020	Merrimack college quarantines 266 residents of a dorm after 17 cases of COVID-19 were discovered in that dorm
01 October 2020	Students in public schools with highest needs can return to school in person
15 December 2020	Universities in Middlesex County begin winter break with start dates ranging from Dec 15th to Dec 23rd
24 December 2020	Winter break for public high schools in Middlesex county
25 January 2021	Universities in Middlesex begin term, with start days from Jan 25th to Mar 1st
15 February 2021	Mid-winter break for public high schools in Middlesex county

References

S1. Coronavirus: Gov. Baker orders schools to remain closed through May 4, ACLU sues ICE to release detainees at risk of COVID-19. *Boston 25 News* [Online]. 25 March 2020. https://www.boston25news.com/news/coronavirus-live-blog-second-mass-resident-dies-covid-19-525-total-cases-across-state/WUF4Z3XGGVDVBES6E3WMOCJYRQ/ (accessed on 10 June 2021).

S2. Office of the Governor for the Commonwealth of Massachusetts. Order requiring face coverings in public places where social distancing is not possible: COVID-19 Order No. 31. Available online: https://www.mass.gov/doc/may-1-2020-masks-and-face-coverings/download (accessed on 10 June 2021).

S3. Here's how Massachusetts will reopen under Governor Charlie Baker's plan. *CBS Boston* [Online]. 18 May 2020. https://boston.cbslocal.com/2020/05/18/massachusetts-reopening-plan-governor-charlie-baker-press-conference-update-businesses-list/ (accessed on 10 June 2021).

S4. New travel order requires quarantine upon entering Massachusetts, includes $500 fine. *CBS Boston* [Online]. 24 July 2020. https://boston.cbslocal.com/2020/07/24/coronavirus-massachusetts-governor-charlie-baker-update-friday-july-24-travel-order-fine-quarantine/ (accessed on 10 June 2021).

S5. Solis S. Citing COVID-19 clusters, compliance violations, Massachusetts postpones second step of Phase 3 reopening 'indefinitely,' Gov. Charlie Baker says. 07 August 2020. https://www.masslive.com/coronavirus/2020/08/citing-covid-19-clusters-compliance-violations-massachusetts-postpones-second-step-of-phase-3-reopening-indefinitely-gov-charlie-baker-says.html (accessed on 10 June 2021).

S6. Creamer L., Kelly, MB. For lower-risk communities only, Mass. loosens some COVID restrictions around gatherings, recreation. *Wbur.* 29 September 2020. https://www.wbur.org/news/2020/09/29/massachusetts-coronavirus-towns-phase-update-october (accessed on 10 June 2021).

S7. Becker, K.M. Mass. pausing indoor ice hockey for 2 weeks due to increasing COVID-19 cases. *10 Boston* [Online]. 23 October 2020. https://www.nbcboston.com/news/coronavirus/mass-pausing-indoor-ice-hockey-for-2-weeks-due-to-increasing-covid-19-cases/2216708/ (accessed on 10 June 2021).

S8. Baker orders curfew for businesses & gatherings, reduces gathering limits, tightens mask mandate. *CBS Boston* [Online]. 02 November 2020. https://boston.cbslocal.com/2020/11/02/massachusetts-coronavirus-reopening-gov-charlie-baker-covid-19-latest-news/ (accessed on 10 June 2021).

S9. Office of the Governor for the Commonwealth of Massachusetts. Order temporarily applying further capacity restrictions to statewide COVID-19 safety rules: COVID-19 Order No. 59. Available online: https://www.mass.gov/doc/covid-19-order-59/download (accessed on 10 June 2021).

S10. Staff Reports. Relaxed COVID restrictions take effect in Massachusetts. *10 Boston* [Online]. 25 January 2021. https://www.nbcboston.com/news/local/relaxed-covid-restrictions-take-effect-in-massachusetts/2286148/ (accessed on 10 June 2021).

S11. City of Somerville. COVID-19 Update for 4/28/20 and free COVID-19 testing in Somerville. Available online: https://www.somervillema.gov/news/covid-19-update-42820-and-free-covid-19-testing-somerville (accessed on 10 June 2021).
S12. Murphy, M. 'Stop the Spread' initiative will increase testing capacity in eight Mass. towns. *Wbur* [Online]. 08 July 2020. https://www.wbur.org/news/2020/07/08/stop-the-spread-testing-initiative (accessed on 10 June 2021).
S13. First responders to receive shots in Massachusetts. *WYNT.com* [Online]. 05 January 2021. https://wnyt.com/capital-region-comeback/first-responders-coronavirus-vaccine-massachusetts/5969081/?cat=10114 (accessed on 10 June 2021).
S14. Carraggi, M. Gillette stadium opening as mass vaccination site: Patch PM. *Patch* [Online]. 12 January 2021. https://patch.com/massachusetts/boston/gillette-stadium-opening-mass-vaccination-site-patch-pm (accessed on 10 June 2021).
S15. Kali, B. COVID mass vaccination site opening at Springfield's Eastfield Mall on Jan. 29, Gov. Charlie Baker says. *Mass LIVE*. [Online]. 25 January 2021. https://www.masslive.com/coronavirus/2021/01/covid-vaccine-site-opening-at-springfields-eastfield-mall-on-jan-29-gov-charlie-baker-says.html (accessed on 10 June 2021).
S16. Fenway Park mass COVID-19 vaccination site now open. *WCVB5.* [Online]. 02 February 2021. https://www.wcvb.com/article/mass-covid-19-vaccination-site-at-fenway-park-set-for-grand-opening-february-1-2021/35375178 (accessed on 10 June 2021).
S17. Staff Reports. Massachusetts enters Phase 2 of vaccination plan; injections begin at Fenway. *10 Boston* [Online]. 01 February 2021. https://www.nbcboston.com/news/local/massachusetts-enters-phase-2-of-vaccination-plan-injections-begin-at-fenway/2290055/ (accessed on 10 June 2021).
S18. Staff Reports. Cleanup underway after storm slams region; some areas see 2 feet of snow. *10 Boston* [Online]. 02 February 2021. https://www.nbcboston.com/news/local/watch-live-noreaster-leaves-thousands-without-power-tuesday-morning/2291036/ (accessed on 10 June 2021).
S19. Germano, B. Mass COVID vaccination site opens at DoubleTree Hotel in Danvers. *CBS Boston* [Online]. 03 February 2021. https://boston.cbslocal.com/2021/02/03/massachusetts-mass-covid-vaccination-sites-danvers-doubletree-hilton-hotel/ (accessed on 10 June 2021).
S20. Staff Reports. Natick Mall opens as latest Mass COVID vaccination site. Here's what to know. *10 Boston* [Online]. 22 February 2021. https://www.nbcboston.com/news/local/a-mass-covid-vaccination-site-has-opened-at-the-natick-mall-heres-what-to-know/2309166/ (accessed on 10 June 2021).
S21. Cote, J. Former Circuit City in Dartmouth opens as newest super COVID vaccination site in Massachusetts. *Mass LIVE* [Online]. 24 February 2021. https://www.masslive.com/coronavirus/2021/02/former-circuit-city-in-dartmouth-opens-as-newest-super-covid-vaccination-site-in-massachusetts.html (accessed on 10 June 2021).
S22. Markos, M., Saperstone, J., Fortier, M. Baker says teachers in Mass. will be eligible to receive vaccine beginning March 11. *10 Boston* [Online]. https://www.nbcboston.com/news/local/baker-to-give-covid-vaccine-update-as-cvs-lists-teachers-as-eligible-in-mass/2318130/ (accessed on 10 June 2021).
S23. New Mass vaccination site at Hynes Convention Center opens. *Wbur* [Online]. 18 March 2021. https://www.wbur.org/commonhealth/2021/03/18/hynes-boston-coronavirus-vaccine-site (accessed on 10 June 2021).
S24. Boston University. Official Academic Calendars. Available online: https://www.bu.edu/reg/calendars/ (accessed on 10 June 2021).
S25. Office of the Governor for the Commonwealth of Massachusetts. Order temporarily closing all public and private elementary and secondary schools. Available online: https://www.mass.gov/doc/march-15-2020-school-closure-order/download (accessed on 10 June 2021).
S26. Office of the Governor for the Commonwealth of Massachusetts. Order temporarily closing all child care programs and authorizing the temporary creation and operation of emergency child care programs. Available online: https://www.mass.gov/doc/march-18-2020-early-education-and-care-order/download (accessed on 10 June 2021).
S27. Lowell Public Schools. 2019-2020 school year calendar. Available online: https://www.lowell.k12.ma.us/cms/lib/MA01907636/Centricity/Domain/4/2019-2020%20School%20Calendar%20with%20Early%20Release%201.pdf (accessed on 10 June 2021).
S28. Lowell Public Schools. 2020-2021 school year calendar. Available online: https://www.lowell.k12.ma.us/cms/lib/MA01907636/Centricity/Domain/30/Approved%20Calendar.pdf (accessed on 10 June 2021).
S29. Town of Framingham School Department. 2019-2020 district calendar. Available online: https://www.framingham.k12.ma.us/cms/lib/MA01907569/Centricity/Domain/81/2018-2019%20SY%20Meetings/02.06.19/Calendar%20English.pdf (accessed on 10 June 2021).
S30. Lexington Public Schools. 2019-2020 school calendar. Available online: https://drive.google.com/file/d/1oJeQRtXWA_4gaGeNiZVAb8Qw9C3YEO9y/view (accessed on 10 June 2021).
S31. Lexington Public Schools. 2020-2021 academic calendar. Available online: https://drive.google.com/file/d/0B971NdYzXEhucGNodmpiWXNlSFVtVXNyM0hpZjNBYzh4aUpR/view (accessed on 10 June 2021).
S32. Greater Lowell Technical School. 2019-2020 PN school calendar. Available online: http://www2.gltech.org/fileBank/CALENDAR%202019-2020.pdf (accessed on 10 June 2021).

S33. Greater Lowell Technical School. 2020-2021 school calendar. Available online: https://www.gltech.org/cms/lib/MA01930064/Centricity/Domain/57/2020-2021%20Calendar_REVISED%20COVID_FINAL%20Approved%20SC3.pdf (accessed on 10 June 2021).
S34. Newton public schools. 2019-2020 school calendar. Available online: https://www.newton.k12.ma.us/cms/lib/MA01907692/Centricity/Domain/4/2019-20%20CALENDAR%20AND%20OBSERVANCES.pdf (accessed on 10 June 2021).
S35. Newton public schools. 2020-2021 school calendar. Available online: https://www.newton.k12.ma.us/cms/lib/MA01907692/Centricity/shared/calendars/2020-21%20CALENDAR.pdf (accessed on 10 June 2021).
S36. Cambridge Rindge and Latin School. 2019-2020 school calendar. Available online: https://crls.cpsd.us/UserFiles/Servers/Server_3045299/File/families/YearinViewCalendar1920.pdf (accessed on 10 June 2021).
S37. Cambridge Public Schools. 2020-2021 district calendar. Available online: https://www.cpsd.us/UserFiles/Servers/Server_3042785/File/cps_district_calendar_2020_21.pdf (accessed on 10 June 2021).
S38. Elizabeth Public Schools. 2020-2021 district calendar. Available online: https://4.files.edl.io/0b9e/10/16/20/162330-57d404f9-6c67-4047-aed3-ec7e6839138b.pdf (accessed on 10 June 2021).
S39. Newton Public Schools. 2019-2020 school calendar. Available online: https://www.newton.k12.ma.us/cms/lib/MA01907692/Centricity/Domain/4/2019-20%20CALENDAR%20AND%20OBSERVANCES.pdf (accessed on 10 June 2021).
S40. Newton Public Schools. 2020-2021 school calendar. https://www.newton.k12.ma.us/cms/lib/MA01907692/Centricity/shared/calendars/2020-21%20CALENDAR.pdf (accessed on 10 June 2021).
S41. Malden Public Schools. 2019-2020 school calendar. Available online: https://maldenps.org/wp-content/uploads/2019/07/2019-2020-DISTRICT-Calendar-FINAL.pdf (accessed on 10 June 2021).
S42. Malden High School. 2019-2020 master calendar. Available online: https://maldenps.org/high/student-life/master-calendar/ (accessed on 10 June 2021).
S43. Malden Public Schools. 2020-2021 school calendar. Available online: https://maldenps.org/blog/2020/03/03/malden-public-schools-2020-2021-school-calendar/ (accessed on 10 June 2021).
S44. Acton-Boxborough Regional School District. School calendar, 2020-2021. Available online: https://www.abschools.org/UserFiles/Servers/Server_216027/File/Calendar/ABRSD%20School%20Calendar%202020-2021%20voted%201-9-20.pdf (accessed on 10 June 2021).
S45. Acton-Boxborough Regional School District. School calendar, 2019-2020. Available online: https://www.abschools.org/UserFiles/Servers/Server_216027/File/Calendar/ABRSD%20School%20Calendar%202019-2020%20VOTED%2012-13-18.pdf (accessed on 10 June 2021).
S46. Harvard University. Ten year calendar. Available online: https://registrar.fas.harvard.edu/ten-year-calendar (accessed on 10 June 2021).
S47. University of Massachusetts Lowell. Spring 2020 academic calendar (updated). Available online: https://www.uml.edu/docs/2020_spring_tcm18-291480.pdf (accessed on 10 June 2021).
S48. University of Massachusetts Lowell. Fall 2020 academic calendar. Available online: https://www.uml.edu/docs/fall_2020_tcm18-300466.pdf (accessed on 10 June 2021).
S49. University of Massachusetts Lowell. Fall 2020 academic calendar. Available online: https://www.uml.edu/docs/2021_spring_tcm18-300542.pdf (accessed on 10 June 2021).
S50. Middlesex Community College. Academic calendar 2019-2020. Available online: https://www.middlesex.mass.edu/academiccalendar/Downloads/20192020f.pdf (accessed on 10 June 2021).
S51. Middlesex Community College. Academic year 2020-2021. Available online: https://www.middlesex.mass.edu/academiccalendar/Downloads/ac2021.pdf (accessed on 10 June 2021).
S52. Tufts University. Academic calendars. Available online: https://students.tufts.edu/registrar/what-we-assist/course-registration-and-scheduling/academic-calendars (accessed on 10 June 2021).
S53. Massachusetts Institute of Technology. Key dates: Spring 2020. Available online: https://registrar.mit.edu/sites/default/files/2019-10/Key%20dates%20calendar%20change%20handout.pdf (accessed on 10 June 2021).
S54. Massachusetts Institute of Technology. Academic calendar. Available online: http://catalog.mit.edu/academic-calendar/ (accessed on 10 June 2021).
S55. Framingham State University. 2019-2020 academic calendar. Available online: https://www.framingham.edu/academics/registrar/resources/academic-calendars/2019-2020-academic-calendar (accessed on 10 June 2021).
S56. Framingham State University. 2020-2021 academic calendar. Available online: https://www.framingham.edu/academics/registrar/resources/academic-calendars/2020-2021-academic-calendar (accessed on 10 June 2021).

S57. Lesley University. Academic calendars. Available online: https://lesley.edu/students/academic-resources/academic-calendars/2020-2021-academic-calendar (accessed on 10 June 2021).
S58. Lesley University. Projected academic calendar dates. Available online: https://lesley.edu/students/academic-resources/academic-calendars/projected-academic-calendar-dates (accessed on 10 June 2021).
S59. Brandeis University. Spring 2020 academic calendar. Available online: https://www.brandeis.edu/registrar/calendar/spring-2020.html (accessed on 10 June 2021).
S60. Brandeis University. Fall 2020 academic calendar. Available online: https://www.brandeis.edu/registrar/calendar/fall-2020.html (accessed on 10 June 2021).
S61. Brandeis University. Spring 2021 academic calendar. Available online: https://www.brandeis.edu/registrar/calendar/spring-2021.html (accessed on 10 June 2021).
S62. University of Massachusetts Amherst. 2019-2020 academic calendar. Available online: https://d2f5upgbvkx8pz.cloudfront.net/sites/default/files/inline-files/2019-20-academic-calendar_6_0.pdf (accessed on 10 June 2021).
S63. Bentley University. Academic calendar Fall 2020. Available online: https://d2f5upgbvkx8pz.cloudfront.net/sites/default/files/inline-files/Academic%2BCalendar%2BEdits%2BFall%2B2020-v4_112420_0.pdf (accessed on 10 June 2021).
S64. Bentley University. Academic calendar Spring and Summer 2021. Available online: https://d2f5upgbvkx8pz.cloudfront.net/sites/default/files/inline-files/AA.503.21%20Spring%20Summer%202021%20Acad%20Cal%20R2_012921.pdf (accessed on 10 June 2021).
S65. Cambridge College. Academic catalog 2019-2020. Available online: https://www.cambridgecollege.edu/sites/default/files/file_uploads/2019-2020catalog-finalwithcover-082619.pdf (accessed on 10 June 2021).
S66. Cambridge College. Academic calendar. Available online: https://www.cambridgecollege.edu/academic-calendar (accessed on 10 June 2021).
S67. Office of the Governor for the Commonwealth of Massachusetts. Order authorizing the reopening of child care programs and rescinding eight COVID-19 Orders. Available online: https://www.mass.gov/doc/signed-rescission-and-childcare-reopen-order-41/download (accessed on 10 June 2021).
S68. Staff and Wire Reports. More than 20 coronavirus cases tied to college party in Worcester. *10 Boston* [Online]. 30 August 2020. https://www.nbcboston.com/news/coronavirus/more-than-20-coronavirus-cases-tied-to-college-party-in-worcester/2186558/ (accessed on 10 June 2021).
S69. Hamm, N. Move-in has begun at Mass. schools with new COVID-19 guidelines. *10 Boston* [Online]. 16 August 2020. https://www.nbcboston.com/news/local/move-in-begun-massachusetts-schools-new-covid-19-guidelines/2178309/ (accessed on 10 June 2021).
S70. Becker, K.M., Johnson M., Buja, M. 266 Merrimack College students from same dorm quarantining after multiple positive COVID tests. *10 Boston* [Online]. 22 September 2020. https://www.nbcboston.com/news/coronavirus/merrimack-college-quarantines-5-students-in-dorm-after-testing-positive-for-covid/2199650/ (accessed on 10 June 2021).
S71. DeCosta-Klipa, N. Boston Public Schools will begin the year fully remote under new 4-phase plan. *Boston.com* [Online]. 21 August 2020. https://www.boston.com/news/education/2020/08/21/boston-public-schools-reopening-plan-remote-start (accessed on 10 June 2021).
S72. Office of the Governor for the Commonwealth of Massachusetts. Order further extending application of additional capacity restrictions to statewide COVID-19 safety rules and rescinding early closing requirements. Available online: https://www.mass.gov/doc/covid-19-order-62/download (accessed on 10 June 2021).
